# Stress levels, hematological condition, and productivity of plasma-producing horses used for snake antivenom manufacture: A comparison of two industrial bleeding methods

**DOI:** 10.1016/j.toxcx.2024.100212

**Published:** 2024-10-21

**Authors:** Ana Margarita Arias-Esquivel, Edwin Moscoso, Deibid Umaña, Mauricio Arguedas, Daniela Solano, Gina Durán, Aarón Gómez, José María Gutiérrez, Guillermo León

**Affiliations:** a Escuela de Zootecnia, Facultad de Ciencias Agroalimentarias, Universidad de Costa Rica, Costa Rica; b Instituto Clodomiro Picado, Facultad de Microbiología, Universidad de Costa Rica, San José, Costa Rica

**Keywords:** Animal welfare, Cortisol, Ethogram, Horse bleeding, Snake antivenom

## Abstract

The immunization and industrial bleeding of horses are essential stages for producing snake antivenoms. In Costa Rica, the traditional method involves stimulating the antibody response of horses by periodically injecting venoms, collecting hyperimmune plasma over three consecutive bleeding days, and repeating this process every eight weeks. While this method does not cause major physical or hematological issues in horses, the associated stress has not been evaluated. We compared this traditional method with an alternative method that involves injecting venoms, collecting hyperimmune plasma in a single bleeding day, and repeating the process every two weeks. We assessed stress (via serum and fecal cortisol levels and an ethological study), hematological parameters (hematocrit and hemoglobin concentration), and plasma productivity over eight months. Serum cortisol levels remained within the normal range for both methods throughout the immunization/bleeding cycle. However, serum and fecal cortisol levels were significantly higher in horses subjected to the traditional method compared to those in the alternative method. Neither method caused significant hematological alterations. Notably, the alternative method yielded a higher volume of plasma. We concluded that adopting the alternative method ensures horse welfare while improving industrial bleeding productivity. This approach may reduce costs and improve the availability of this essential treatment for vulnerable populations.

## Highlights

• Antivenom manufacturers must ensure the welfare of horses used as immunoglobulin source.
• The immunization, bleeding, and care of horses must be continuously reviewed and improved.
• A high-intensity, low-frequency bleeding method was compared to a low-intensity, high-frequency method regarding stress, hematologic and yield parameters.
• The low-intensity, high-frequency method achieved higher plasma yield while ensuring horse welfare.

## Graphical abstract

**Figure:**
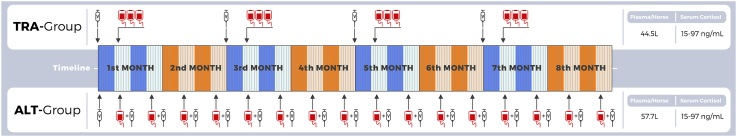

## 1 Introduction

Snakebite envenomation is an important public health concern that affects millions of people and domestic animals worldwide, especially in rural regions of sub-Saharan Africa, Asia, and Latin America (Afroz et al., 2024). The mainstay in the therapy of snakebite envenomation is the prompt administration of antivenoms (WHO, 2017), which are formulations of immunoglobulins purified from the plasma of animals, mainly horses or sheep, that have been immunized against snake venoms (León et al., 2018).

The immunization process involves the repeated injection of snake venoms mixed with immunological adjuvants that enhance the animals’ antibody response. The anti-venom antibodies are then harvested through the collection of blood plasma (León et al., 2011). In the case of horses, the traditional method used in Costa Rica for obtaining plasma involves collecting 6–8 L of blood/day/animal for three consecutive days, with an 8-week interval between bleeding cycles. After sedimentation and plasma separation, the red blood cells (RBCs) are resuspended in saline solution and transfused back into the corresponding horse to reduce the risk of anemia. Following a rest period of seven weeks, the animals are re-immunized with the venoms and subjected to industrial bleeding again (Vaz and Araujo, 1949; Angulo et al., 1997; Huertas et al., 2023).

It has been demonstrated that the traditional procedures of immunization and bleeding used in Costa Rica do not result in significant health issues for horses (Angulo et al., 1997; Huertas et al., 2023). However, the stress experienced by the animals during these procedures has not been evaluated. Ensuring that animals are not exposed to conditions that favor physical stress (e.g., hunger, thirst, fatigue, extreme environmental conditions, injuries) or psychological stress (e.g., aggressive handling, space restriction, loud noises) during immunization, industrial bleeding, and general husbandry is an essential ethical concern in antivenom production.

When subjected to inadequately designed procedures, horses may feel threatened and become stressed. This can activate the hypothalamic-pituitary-adrenal (HPA) axis and induce the release of cortisol into the bloodstream (Cerasoli et al., 2022). Therefore, the cortisol concentrations in serum, saliva, or feces have been widely used in the study of stress experienced by horses under various management conditions (Sauer et al., 2019; Cerasoli et al., 2022; Olvera-Maneu et al., 2023). In addition, stress in horses can be evidenced by changes in the occurrence, frequency, or intensity of discomfort manifestations that contrast with the normal behavioral pattern (Torcivia and McDonnell, 2021). Therefore, ethological indicators have also been used to improve the welfare of horses used for different purposes (Dyson and Pollard, 2022; Potier and Louzier, 2023; Dyson and Pollard, 2024).

In this study, a group of horses subjected to the traditional bleeding method (TRA-group) was compared to a group of horses subjected to an alternative, less intensive method (ALT-group) in terms of stress response (serum and fecal levels of cortisol, and ethological characterization), hematological condition (hematocrit and hemoglobin concentration), and productivity of plasma for snake antivenom manufacture.

As the ALT-group was not exposed to blood collections carried out on consecutive days, we hypothesized that the stress and hematological demands experienced by these horses would be lower than those experienced by horses in the TRA-group. The results of this study could help improve animal welfare and the ethical performance of snake antivenom production, as well as enhance the productivity of this industrial activity.

## 2 Materials and methods

### 2.1 Ethical considerations

All procedures used in this study were approved by the Institutional Committee for the Care and Use of Laboratory Animals (CICUA) of Universidad de Costa Rica (Proceedings 82-08 and 39-20) and comply with the International Guiding Principles for Biomedical Research Involving Animals (CIOMS, 1985). These procedures were carried out following appropriate animal welfare protocols to ensure the well-being of the horses and to minimize any potential distress or harm. Groups of horses were herded in paddocks located at an elevation of 1495 m above sea level, with access to pasture and water available *ad libitum*. The horses were also supplemented with pelleted feed enriched with proteins, vitamins, and minerals. Immunization and bleeding procedures were carried out under the supervision of a veterinarian in a facility specifically designed for these purposes, located on the same farm where the horses were kept.

### 2.2 General characteristics of horses

A total of 48 horses of undefined breed (350–450 kg), immunized against the venoms of *Bothrops asper*, *Crotalus simus*, *Crotalus pifanorum*, and *Lachesis stenophrys*, were used. Over the last three years, these horses underwent the traditional immunization/bleeding method (see section 2.3.) for the routine production of PoliVal-ICP antivenom. They were then randomly divided into two groups: the TRA-group (consisting of 22 horses, 5 males and 17 females, aged 14 ± 2 years, 444 ± 53 kg of body weight, and a body condition score (BCS) of 5.4 ± 1.5), which continued with the traditional immunization/bleeding method, and the ALT-group (consisting of 26 horses, 12 males and 14 females, aged 14 ± 5 years, 421 ± 70 kg of body weight, and a BCS of 5.7 ± 1.7), which switched to an alternative, less intensive immunization/bleeding method (see section 2.4.). During the following eight months, these groups of horses were compared at different stages of the plasma production process regarding their hematological condition and the productivity of hyperimmune plasma. Additionally, in the eighth month, a comparison of stress levels experienced by the horses in both groups was conducted.

### 2.3 Traditional immunization/bleeding method

Every eight weeks, the horses in the TRA-group were reimmunized with a venom mixture composed of 2 mg B*. asper* venom, 1 mg C*. simus* venom, and 1 mg C*. pifanorum* venom, dissolved in 4 mL of sterile saline solution. Every three reimmunizations, 2 mg L*. stenophrys* venom was included in the venom booster (Arroyo et al., 2017; Alfaro-Chinchilla et al., 2021). Ten days after the reimmunization booster, a physical evaluation was performed to ensure that only horses in optimal condition were subjected to industrial bleeding. Industrial bleeding was performed over three consecutive days (i.e., days 12, 13, and 14 after the reimmunization). On the first day, a system of PVC blood bags was used to collect 6–8 L of blood from the jugular vein of each horse. Citrate dextrose solution (ACD; containing citric acid 0.093 mol/L, sodium citrate 0.197 mol/L, and dextrose 0.6 mol/L) was used as anticoagulant. The blood was stored at 2–8 °C overnight to allow the RBCs to sediment. On the second day, the plasma was separated, preserved with the addition of 0.005% thimerosal, and stored at 2–8 °C. Meanwhile, the RBCs were resuspended in 3 L of saline solution (0.15 M NaCl) and warmed to 37 °C. On that day, horses underwent another cycle of 6–8 L blood collection, followed by self-transfusion of the RBCs collected on the first day. Again, the blood was stored at 2–8 °C overnight. On the third day, the sedimented RBCs were separated from the plasma, resuspended in saline solution, and warmed to 37 °C. An additional 6–8 L of blood were collected, and the horses underwent self-transfusion of the RBCs collected on the second day. The RBCs collected on the third day were not transfused back to the horses (Vaz and Araujo, 1949; Angulo et al., 1997b; Huertas et al., 2023). Following this, the horses were given a 7-week rest period before being subjected to reimmunization, physical evaluation and industrial bleeding again, as described. One immunization/bleeding cycle was defined as the total number of immunizations and bleedings conducted over an eight-week period. For the TRA-group, this corresponds to one immunization and three consecutive bleeding days. During the last cycle, the stress levels of the horses at various stages of the plasma production process were also assessed.

### 2.4 Alternative immunization/bleeding method

Every two weeks, the horses in the ALT-group were reimmunized by the SC injection of a venom mixture composed of 0.2 mg B*. asper* venom, 0.1 mg C*. simus* venom, and 0.1 mg C*. pifanorum* venom, dissolved in 4 mL of sterile saline solution. Every nine reimmunizations, 0.2 mg L*. stenophrys* venom was included in the venom booster (Arroyo et al., 2017; Alfaro-Chinchilla et al., 2021). Ten days after the first reimmunization booster, the horses that were in good condition as judged by the physical examination were subjected to industrial bleeding, with 6–8 L of blood collected per horse in a single day. The blood was anticoagulated with ACD and stored at 2–8 °C. Immediately after bleeding the horses were reimmunized with the venom mixture described above. Two weeks later, the sedimented RBCs were separated from the plasma, suspended in 3 L of saline solution, and warmed to 37 °C. The horses then underwent another industrial bleeding. Immediately after collecting 6–8 L of blood, the horses were transfused with their own resuspended RBCs from the bleeding performed two weeks before and were then reimmunized. One immunization/bleeding cycle was defined as the total of immunizations and bleedings conducted in an eight-week period in order to compare it with the traditional bleeding protocol. For the ALT-group, this corresponds to four immunizations and four bleeding days at 2-week intervals. During the fourth cycle, the stress levels of the horses were evaluated at different points during the plasma production process.

### 2.5 Assessment of stress levels in plasma-producing horses

The horses were submitted for eight months to several cycles of immunization and bleeding, as described above. In the last cycle of immunization/bleeding, the stress levels of horses were evaluated. For this, blood and fecal samples were collected at four points during the last cycle, i.e., before immunization, immediately after immunization with venom, ten days after immunization, and after bleeding. Blood samples (10 mL) were drawn from the jugular vein of each horse using Vacutainer® EDTA tubes for hematology tests and Vacutainer® serum tubes for cortisol quantification. In addition, single fecal samples were collected from the rectum of each horse 16–18 h. The samples were stored at −20 °C in individual airtight plastic bags. Fecal samples were subsequently processed to obtain fecal liquid. Feces were thoroughly mixed, and 0.5 g was wrapped in two layers of cheesecloth and manually compressed to release fecal liquid into a glass beaker. A transfer pipette was used to transfer fecal liquid to 1 ml plastic vials. The samples were then centrifuged for 15 min at 3000×*g*, to remove the solids, and the supernatant liquid was collected for analysis.

#### 2.5.1 Quantification of cortisol

Cortisol concentration in both serum and feces was quantified using the MAGLUMI cortisol (CLIA) assay (Shenzhen New Industries Biomedical Engineering Co., Ltd., China) in a fully automated chemiluminescence immunoassay (CLIA) analyzer, MAGLUMI 2000 ® (Shenzhen New Industries Biomedical Engineering Co., Ltd, China). It was considered that the normal range of serum cortisol in horses is 15–97 ng/mL (Larsson et al., 1979).

#### 2.5.2 Ethological study

Behavioral variables were assessed after the bleeding at the end of the last immunization/bleeding cycle using an ethogram developed based on previous studies (Anderson et al., 2015; Hintze et al., 2016; Burn, 2017). To eliminate the influence of foreign presence that could affect the evaluation, the observation was conducted avoiding interactions between horses and observers. Five random horses per group were individually assessed during the industrial bleeding process. The behavioral variables assessed, and their corresponding score ranges are listed in Table 1.

**Table 1:** Categorization of behaviors based on their occurrence, frequency, and intensity.

Behavior	Intensity/frequency score			
	1	2	3	4
Kicking	Standing quietly, no kicking	Occasional (1–2 times/5 min)	Frequent (3–4 times/5 min)	Excessive (>5 times/5 min)
Scratching the ground	No scratching	Occasional (1–2 times/5 min)	Frequent (3–4 times/5 min)	Excessive (>5 times/5 min)
Head movement	No signs of discomfort	Intermittent (1–2 times/5 min)	Rapid intermittent (3–4 times/5 min)	Continuous (>5 times/5 min)
Eye sclera visibility	Sclera not visible	Occasional (1–2 times/5 min)	Moderate (3–4 times/5 min)	Frequently (>5 times/5 min)
Eye wrinkles	No visible	Soft present	Prominent present	
Vocalizations	No vocalizations	Few (1–2 times/5 min)	Moderate (3–4 times/5 min)	Many (>5 times/5 min)
Ears back	Not present	Moderately present	Obviously present	
Prominent jaw	Not present	Moderately present	Obviously present	
Tense mouth	Not present	Moderately present	Obviously present	
Tense nostrils	Not present	Moderately present	Obviously present	
Closed eyelids	Not present	Moderately present	Obviously present	
Tension above the eye area	Not present	Moderately present	Obviously present	
Flehmen response	No Flehmen response	Occasional (1–2 times/5 min)	Frequent (3–4 times/5 min)	Excessive (>5 times/5 min)
Yawning	No yawning	Occasional (1–2 times/5 min)	Frequent (3–4 times/5 min)	Excessive (>5 times/5 min)
Urination	Not present (no urination)	Moderate (once)	Obvious (more than once)	
Defecation	Not present (no defecation)	Moderate (once)	Obvious (more than once)	
Chewing-like movements	Not present	Occasional (1–2 times/5 min)	Frequent (3–4 times/5 min)	Excessive (>5 times/5 min)

### 2.6 Physical and hematological evaluation of plasma-producing horses

Along the whole eight-month period of repeated cycles of immunization and bleeding, physical and hematological evaluations were conducted in an immunization/bleeding facility specifically designed for these activities. Horses were examined by a veterinarian to determine the presence of wounds, lesions, signs of illness, and the development of diffuse edema, soft abscesses, or solid fibrous tissue resulting from immunization (Arguedas et al., 2022). The horses’ physical condition was assessed using the Hennecke body condition scale (BCS; Henneke et al., 1983), which ranges from 1 (extremely emaciated) to 9 (extremely fat), with the optimal range being between 4 and 6. For the hematological assessment, 10 mL blood samples were obtained from the jugular vein of each horse using Vacutainer® EDTA tubes. Hematocrit and hemoglobin concentrations were analyzed using a Veterinary Hematology Analyzer (BC-5000 Vet; Mindray Animal Care, Shenzhen, China).

### 2.7 Determination of plasma productivity

The plasma productivity was determined by calculating the total volume of plasma produced by each horse during an immunization/bleeding cycle (8-week period). In the case of the TRA-group, this corresponds to one immunization and three consecutive bleeding days in the 8-week period, while for the ALT-group this corresponds to four immunizations and four bleeding days in an 8-week period. Plasma productivity was calculated for a total of four immunization/bleeding cycles for both groups.

### 2.8 Total protein content and neutralization ability of antivenoms

Antivenom batches produced from plasma collected with the traditional method (batches 6580121POLQ, 6660521POLQ and 6680621POLQ) or from plasma collected with the alternative method (batches 6910123POLQ, 6940323POLQ and 6930223POLQ) were analyzed. The total protein content in the antivenoms was quantified using the Biuret method (Gornall et al., 1949). The efficacy of antivenoms in neutralizing the lethality of snake venoms was evaluated using a mouse model. Groups of five CD-1 mice (both male and female, weighing 16–18 g) received an intraperitoneal injection of 0.5 mL of various dilutions of antivenom, combined with a fixed challenge dose of venom (4 LD_50_s), using phosphate-buffered saline (PBS) as solvent. The mixtures were incubated at 37 °C for 30 min prior to injection. Control groups of mice received mixtures in which the antivenom was replaced by PBS. The number of deaths within the subsequent 48 h was recorded to determine the median effective dose (ED_50_) and its associated 95% confidence interval (95% CI) using Probit analysis (Finney, 1971). The ED_50_ values were expressed as milligrams of venom neutralized per gram of antivenom. The surviving mice were euthanized by CO_2_ inhalation.

### 2.9 Statistical analysis

Comparisons of serum and fecal cortisol levels at various stages of the immunization/bleeding methods, as well as between horse groups at each stage, were assessed using an independent *t*-test. Similarly, the differences in plasma productivity between the two horse groups and the ED_50_ values of antivenom batches produced from plasma collected using both bleeding methods were evaluated using an independent *t*-test. Differences in the behaviors and in hematocrit and hemoglobin were assessed by a Mann-Whitney *U* test. Variations in hematocrit and hemoglobin values through cycles for the ALT-group were tested by a general linear model of repeated measures. P-values <0.05 were considered statistically significant. All the tests were conducted using IBM SPSS (IBM Corp. Released, 2020. IBM SPSS Statistics for Windows, Version 27.0. Armonk, NY: IBM Corp).

## 3 Results and discussion

### 3.1 Assessment of stress levels in plasma-producing horses

Animal welfare is a concern that has led Instituto Clodomiro Picado (ICP) to the permanent improvement of the procedures involving the animals used for antivenom production. The horses in this study were herded in groups with access to pasture and water *ad libitum*, so interferences by stress caused by social isolation or restriction of forage intake (Mal et al., 1991; McGreevy et al., 1995) were not expected. Moreover, conducting immunization and bleeding in a specialized facility on the same property where the horses were routinely herded helps to prevent stress associated with transportation (Miller et al., 2021). Consequently, it is assumed that the stress analyzed in this study was mainly caused by the immunization and bleeding processes themselves.

**Fig. 1:**
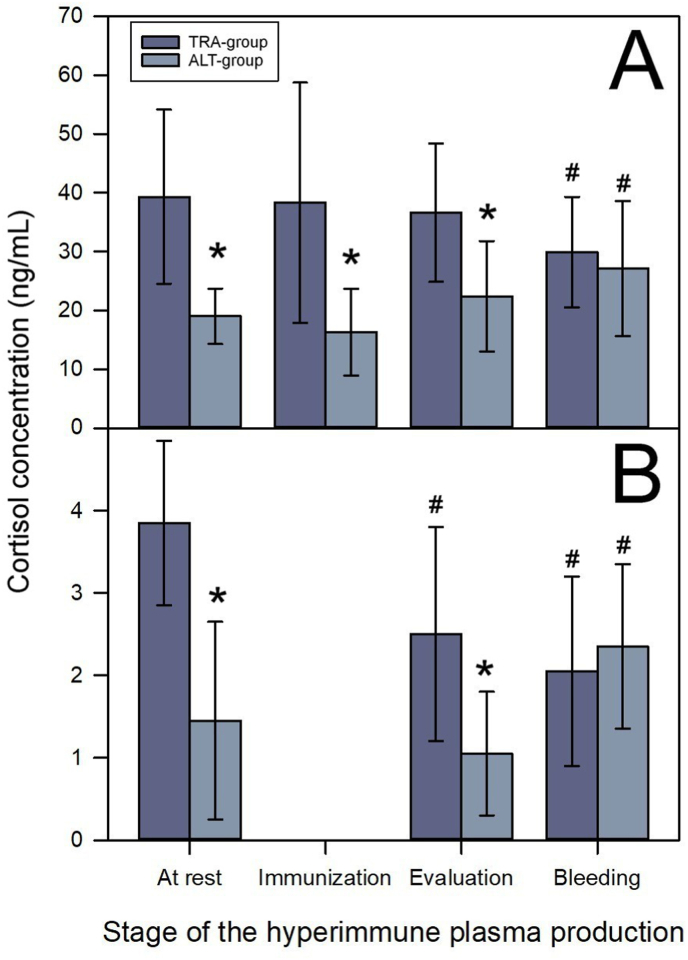
Cortisol concentrations in serum (A) or feces (B) of horses subjected to the traditional (TRA-group) or the alternative (ALT-group) immunization/bleeding method. Samples were collected at different stages of the plasma production process: At rest (ten days before the end of the rest period), Immunization (immediately after venom injection), Evaluation (during the evaluation of the physical and hematological conditions of horses, 10 days after venom injection), and Bleeding (immediately after the end of the third bleeding day in the traditional method, or the single bleeding day in the alternative method). ∗*p* < 0.05 in the comparison between both groups of horses at the same production stage. ^#^*p* < 0.05 in the comparison between different production stages and samples collected at rest, for the same group of horses. The normal range of serum cortisol levels in horses is 15–97 ng/mL (Larsson et al., 1979).

After seven months of being subjected to either the traditional or the alternative immunization/bleeding methods, and before the onset of the last cycle of immunization/bleeding, horses in both groups showed cortisol levels within the normal range (i.e., 15–97 ng/mL; Larsson et al., 1979). However, the cortisol levels in the TRA-group were significantly higher than those in the ALT-group in both serum (Fig. 1A; *t*_(30.885)_ = 6.626; *p* < 0.001) and fecal samples (Fig. 1B; *t*_(40)_ = 6.929; *p* < 0.001). Thus, even though cortisol levels were within the normal ranges in horses in both groups, the fact that there was a significantly higher concentration of cortisol in the TRA-group suggests that these horses had a higher level of stress as compared to horses of the ALT-group before the last immunization scheme.

One week later, the horses were reimmunized with the mixture of venoms. Reimmunization in some horses can lead to lesions with different clinical manifestations, such as diffuse edema around the injection site, the formation of soft abscesses, or the development of solid fibrous tissue. Occasionally, these lesions can open fistulas through which bloody pus-like material is discharged (Arguedas et al., 2022). All these lesions cause pain, which may stress the horses. Therefore, it was of interest to assess the cortisol levels immediately after venom injection.

Serum cortisol levels in samples collected within 30 min after immunization did not differ from those observed before immunization in both the TRA-group (Fig. 1A; *t*_(50)_ = −0.191; *p* = 0.850) and the ALT-group (Fig. 1A; *t*_(42)_ = −1.455; *p* = 0.153). These findings suggest that the stress induced by the acute effects of venom injection was minimal. Additionally, as observed in samples collected before immunization, the serum cortisol levels were significantly higher in the TRA-group than in the ALT-group (Fig. 1A; *t*_(46)_ = 4.806; *p* < 0.001). This observation suggests that the traditional method is more stressful than the alternative method. The early effects of immunization on fecal cortisol were not assessed.

Ten days after immunization, the horses underwent a physical evaluation to ensure that only those in optimal conditions would be subjected to industrial bleeding. This opportunity was also used to assess the stress caused by the delayed effects of the venom injection. For the TRA-group, the cortisol levels in serum samples collected during the evaluation did not differ from those obtained before immunization (Fig. 1A; serum: *t*_(50)_ = −0.715; *p* = 0.478), but significant differences were found in feces (Fig. 1B; *t*_(44)_ = 3.769; *p* < 0.001). For the ALT-group, no significant differences were found between samples collected during this evaluation and those collected before immunization in both serum (Fig. 1A; *t*_(30.932)_ = 1.510; *p* = 0.141) and feces (*t*_(42)_ = 1.387; *p* = 0.173). These results suggest that the delayed effects (assessed at 10 days) of the venom injection have a minimal impact on the stress experienced by the horses. However, despite the fact that cortisol levels did not differ from those of horses at rest, significant differences were found between the two groups of horses in terms of their cortisol concentrations in serum (Fig. 1A; *t*_(46)_ = 4.582; *p* < 0.001) and in feces (Fig. 1B; *t*_(46)_ = 4.693; *p* < 0.001), again suggesting that horses submitted to the traditional method had higher stress than those submitted to the alternative method.

The analysis of samples collected at the end of the industrial bleeding showed significant differences between the cortisol levels in the samples collected at this time and the samples collected before immunization in the TRA-group in serum (Fig. 1A; *t*_(47)_ = −2.607; *p* = 0.012) and feces (Fig. 1B; *t*_(41)_ = 5.468; *p* < 0.001) and in the ALT-group in serum (Fig. 1A; *t*_(27.878)_ = 3.044; *p* = 0.005) and feces (Fig. 1B; *t*_(42)_ = −2.632; *p* = 0.012). Immediately after bleeding, no significant differences were found between the two groups in terms of serum (Fig. 1A; *t*_(43)_ = 0.922; *p* = 0.362) or fecal cortisol levels (Fig. 1B; *t*_(43)_ = −0.922; *p* = 0.362).

The behavior of horses subjected to the traditional and alternative methods was analyzed during the bleeding using the ethogram described in Table 1. Major abnormal behaviors such as stereotypes, dangerous self-directed behaviors, affective behaviors, or displacement activities were not detected during the industrial bleeding, and only minor changes in the observed parameters were noticed (Table 2). The occurrence, frequency and intensity of the variables described in the ethogram were not significantly different between both groups (Table 2). Thus, ethological observations did not reveal clear evidence of stress in both groups of horses during the last cycle of immunization/bleeding.

**Table 2:** Ethogram of horses undergoing industrial bleeding for plasma collection^a^.

Behavior	TRA-group	ALT-group	Mann-Whitney *U* Test
Kicking	0.0 ± 0.0	0.0 ± 0.0	U: 15.0; *p* = 0.424
Scratching the ground	0.2 ± 0.4	0.4 ± 0.9	U: 13.0; *p* = 1.000
Head movement	1.8 ± 1.3	2.2 ± 1.3	U: 15.0; *p* = 0.654
Eye sclera visibility	0.4 ± 0.5	0.8 ± 1.3	U: 17.0; *p* = 0.366
Eye wrinkles	0.6 ± 0.5	1.0 ± 0.7	U: 16.5; *p* = 0.403
Vocalizations	0.0 ± 0.0	0.0 ± 0.0	U: 12.5; *p* = 1.000
Ears back	1.2 ± 0.4	1.8 ± 0.4	U: 20.0; *p* = 0.093
Prominent jaw	0.2 ± 0.4	0.8 ± 0.4	U: 20.0; *p* = 0.093
Tense mouth	0.2 ± 0.4	0.6 ± 0.5	U: 17.5; *p* = 0.270
Tense nostrils	1.0 ± 0.7	0.6 ± 0.5	U: 8.5; *p* = 0.434
Closed eyelids	0.4 ± 0.5	0.4 ± 0.5	U: 14.0; *p* = 0.796
Tension above the eye area	0.4 ± 0.5	0.4 ± 0.5	U: 13.5; *p* = 0.905
Flehmen response	0.0 ± 0.0	0.0 ± 0.0	U: 12.5; *p* = 1.000
Yawning	0.0 ± 0.0	0.0 ± 0.0	U: 12.5; *p* = 1.000
Urination	0.0 ± 0.0	0.0 ± 0.0	U: 12.5; *p* = 1.000
Defecation	0.0 ± 0.0	0.0 ± 0.0	U: 12.5; *p* = 1.000
Chewing-like movements	1.0 ± 0.7	1.2 ± 1.3	U: 13.0; *p* = 1.000

a Results correspond to the average ± SD (n = 5).

### 3.2 Evaluation of hematological condition of plasma-producing horses

**Fig. 2:**
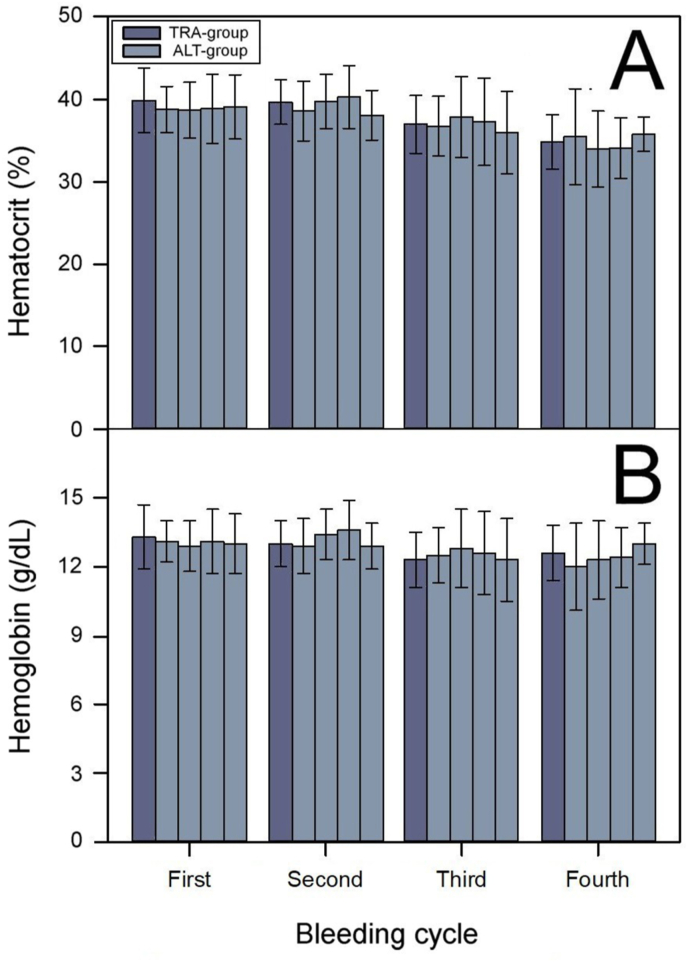
Hematocrit (A) and hemoglobin concentration (B) of horses subjected to the traditional (TRA-group) or the alternative (ALT-group) immunization/bleeding method. Samples were collected at four consecutive immunization/bleeding cycles. The two groups did not show significant differences in terms of hematocrit (Mann-Whitney U: 0.475; *p* = 0.635) or hemoglobin concentration (Mann-Whitney U: 0.032; *p* = 0.975). The normal ranges determined by the Quality Control Laboratory of Instituto Clodomiro Picado for hematocrit and hemoglobin concentration values are 38–50% and 13.3–17.9 g/dL, respectively.

Over four immunization/bleeding cycles, horses in both the TRA-group and the ALT-group had normal values of hematocrit (normal range determined by the Quality Control Laboratory of Instituto Clodomiro Picado: 28–50%; Fig. 2A) and hemoglobin concentration (normal range determined by the Quality Control Laboratory of Instituto Clodomiro Picado: 13.3–17.9 g/dL; Fig. 2B). The two groups did not show significant differences in terms of hematocrit (Mann-Whitney U: 0.475; *p* = 0.635) or hemoglobin concentration (Mann-Whitney U: 0.032; *p* = 0.975). Since the traditional bleeding method does not result in significant hematological alterations (Angulo et al., 1997; Huertas et al., 2023), there was limited potential for the alternative bleeding method to enhance animal welfare in this regard. These results indicate that using the alternative bleeding method for eight months did not improve nor worsen the hematological conditions of the plasma-producing horses.

### 3.3 Comparison of productivity between the two industrial bleeding methods

**Fig. 3:**
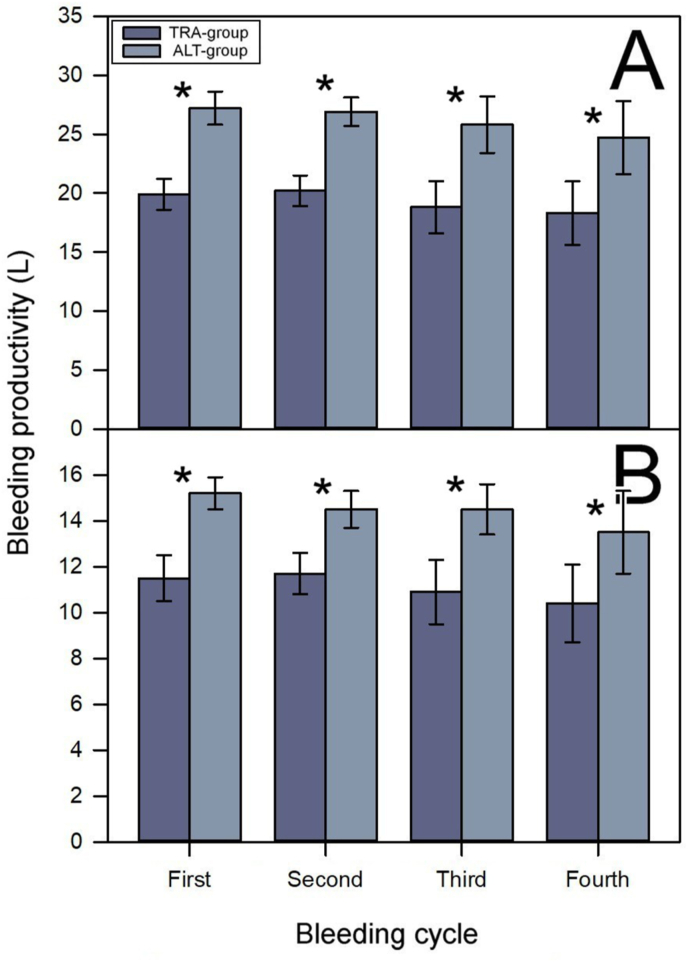
Productivity of blood (A) and hyperimmune plasma (B) from horses subjected to the traditional (TRA-group) or the alternative (ALT-group) immunization/bleeding method. Productivity was measured over four consecutive immunization/bleeding cycles. The productivity of plasma in each cycle was significantly higher in the ALT-group in both blood and plasma. ∗*p* < 0.001.

For every immunization/bleeding cycle, three blood bags were collected from each horse in the TRA-group, while four bags were collected from each horse in the ALT-group. Consequently, the productivity of plasma was significantly higher in the ALT-group in both blood (Fig. 3A) and plasma (Fig. 3B). No significant differences were observed between batches of antivenom produced from plasma collected using the traditional method and those collected via the alternative method in terms of their efficacy in neutralizing the venoms of *B. asper* (Table 3; *t*_(4)_ = −1.976; *p* = 0.119), *C. simus* (Table 3; *t*_(4)_ = −0.763; *p* = 0.488), *C. pifanorum* (Table 3; *t*_(4)_ = −2.662; *p* = 0.056), and *L. stenophrys* (Table 3; *t*_(4)_ = 0.288; *p* = 0.788). These results indicate that the implementation of the alternative method could help improve the efficiency of hyperimmune plasma production, thereby reducing the production costs of antivenoms.

**Table 3:** Comparison of the median effective dose (ED_50_^a^) of antivenom batches produced from plasma collected with different bleeding methods.

	Method of plasma collection	
	Traditional method	Alternative method
ED_50_ anti-*B. asper*^a^	52 ± 4	62 ± 8
ED_50_ anti-*C. simus*^a^	94 ± 19	105 ± 17
ED_50_ anti-*C. pifanorum*^a^	15 ± 1	20 ± 3
ED_50_ anti-*L. stenoprhys*^a^	90 ± 21	69 ± 11
Total protein content (g/dL)	6.5 ± 0.6	5.2 ± 0.3

a The values represent the average ± standard deviation (SD) of the ED_50_ values from three industrial batches of antivenom (n = 3), expressed in milligrams of venom neutralized per gram of antivenom. No significant differences were observed between batches of antivenom produced from plasma collected using both methods in terms of their efficacy in neutralizing the venoms of *B. asper* (*t*_(4)_ = −1.976; *p* = 0.119), *C. simus* (*t*_(4)_ = −0.763; *p* = 0.488), *C. pifanorum* (*t*_(4)_ = −2.662; *p* = 0.056), and *L. stenophrys* (*t*_(4)_ = 0.288; *p* = 0.788).

## 4 Conclusions

Both the traditional and alternative methods of industrial immunization/bleeding resulted in low stress for the horses, as judged by cortisol levels and behavioral parameters. Cortisol levels were higher in the TRA-group than in the ALT-group, but in both cases were within normal levels in horses. Similarly, both methods had minimal impact on the hematological condition of the horses. In contrast, the plasma productivity of the alternative method was significantly higher than that of the traditional method. The insights into the immediate impact of the two bleeding methods suggest that adopting the alternative method of industrial bleeding could be a viable option for enhancing productivity while ensuring the welfare of the horses used as a plasma source for snake antivenom production. However, a more comprehensive exploration of the long-term effects of these bleeding methods on horse health and productivity over extended periods of time is necessary to draw more definitive conclusions. While the development of next-generation antivenoms, based on recombinant antibodies, is gaining momentum, animal-derived antivenoms will continue to play a crucial role in helping vulnerable populations in the treatment of snakebite envenomations. Therefore, enhancing the effectiveness, safety, and affordability of these products remains an ongoing challenge.

## CRediT authorship contribution statement

**Ana Margarita Arias-Esquivel:** Writing – review & editing, Writing – original draft, Investigation, Conceptualization. **Edwin Moscoso:** Writing – review & editing, Investigation. **Deibid Umaña:** Writing – review & editing, Investigation. **Mauricio Arguedas:** Writing – review & editing, Investigation. **Daniela Solano:** Writing – review & editing, Investigation. **Gina Durán:** Investigation, Writing – review & editing. **Aarón Gómez:** Writing – review & editing, Investigation. **José María Gutiérrez:** Writing – review & editing, Writing – original draft, Funding acquisition, Conceptualization. **Guillermo León:** Writing – review & editing, Writing – original draft, Project administration, Funding acquisition, Conceptualization.

## Ethical statement

This manuscript presents an experimental study conducted in accordance with standard scientific ethical procedures, including those concerning the use and welfare of animals.

## Ethical statement

This study was approved by the Institutional Committee for the Care and Use of Laboratory Animals (CICUA) of Universidad de Costa Rica (reference numbers 82-08 and 39-20)

## Declaration of competing interest

The authors declare the following financial interests/personal relationships which may be considered as potential competing interests:

Guillermo Leon reports financial support was provided by 10.13039/100010269Wellcome Trust. If there are other authors, they declare that they have no known competing financial interests or personal relationships that could have appeared to influence the work reported in this paper.

## Data Availability

Data of the results presented in this study will be available from the authors upon reasonable request.
